# Physiological and Histological Responses of Awassi Lambs to High Dietary Organic Copper Supplementation

**DOI:** 10.3390/ani15071066

**Published:** 2025-04-07

**Authors:** Osama A. Saeed, Mohanad A. Shareef, Hassan M. Alnori, Teik K. Leo, Mohammed A. Al-Bayar, Idham A. Abed, Omar K. Attallah

**Affiliations:** 1 Department of Animal Production, College of Agriculture, University of Anbar, Ramadi 31001, Iraq; ag.hassan.alnori@uoanbar.edu.iq (H.M.A.); ag.mohammed.ala@uoanbar.edu.iq (M.A.A.-B.); ag.omar.k.attalah@uoanbar.edu.iq (O.K.A.); 2 Department of Accommodation Affairs, Headquarter, University of Anbar, Ramadi 31001, Iraq; m.a.shareef@uoanbar.edu.iq; 3 Imperium Grp Sdn Bhd, Unit 43-7, The Boulevard, Mid Valley City Lingkaran Syed Putra, Kuala Lumpur 59200, Malaysia; leoteikee@hotmail.com; 4 Department of Soil Sciences and Water Resources, College of Agriculture, University of Anbar, Ramadi 31001, Iraq; ds.dr.idhamalassafii@uoanbar.edu.iq

**Keywords:** copper supplementation, Awassi sheep, physiology, histology, gene expression

## Abstract

This study investigated the effects of high dietary organic copper supplementation on growth performance, mineral metabolism and organ histology in male Awassi lambs. Fifteen lambs with similar body weight (16 ± 1.53 kg) were assigned to three groups: a control group (T1) receiving a basal diet, and two treatment groups (T2 and T3) supplemented with 0.5 g and 1 g of organic copper per kg DM, respectively. The total dietary copper concentrations were 6.18, 81.68 and 156.75 ppm in T1, T2, and T3 groups, respectively. The basal diet, composed of soybeans, wheat, and barley, provided 2.4 Mcal/kg metabolizable energy and 14% crude protein. High dietary copper supplementation had no significant impact on growth performance and body biometric measurements. Testicular length was increased in T3 lambs. Mineral analysis showed increased hepatic copper concentrations in T2 and T3 lambs. Kidney copper levels remained within normal ranges, while renal zinc concentrations declined in T2 and T3 lambs. Gene expression analysis demonstrated upregulation of *ATP7A* and *ATP7B* genes in T3 lambs, indicating a cellular adaptation to elevated copper levels, while *IGF1* expression remained unchanged. Histological assessments, however, revealed hepatic and renal changes in T3 lambs. Overall, high dietary organic copper supplementation, particularly at 1 g Cu/kg DM, increases copper retention and may support reproductive health in Awassi lambs. However, excessive intake poses risks of liver and kidney damage, highlighting the need for careful dietary management.

## Simple Summary

Copper is an essential trace mineral for maintaining optimal reproductive performance and physiological health in sheep. However, sheep are particularly sensitive to copper toxicity. This study investigated the effects of high dietary organic copper supplementation on growth performance and health of Awassi lambs. The results showed that high dietary copper supplementation did not affect growth performance, and the lambs remained healthy. Lambs fed a high-copper diet, particularly at 1 g Cu/kg dry matter (DM), showed increased testicular length, suggesting potential benefits for sperm production without any negative impact on testicular structure. However, we observed signs of potential liver damage alongside elevated hepatic copper levels, though no systemic adverse effects were detected. This may indicate that Awassi sheep possess robust homeostatic mechanisms to regulate copper storage and prevent excessive accumulation in vital organs. These findings are further supported by the increased activity of *ATP7A* and *ATP7B* genes, which mediate copper transport, while the unchanged activity of *IGF1* gene suggests that copper has no impact on growth-regulating pathways. Overall, high dietary organic copper supplementation, particularly at 1 g Cu/kg DM, increases copper absorption and may support reproductive health in Awassi lambs without causing harmful effects.

## 1. Introduction

Sheep farming is a cornerstone of global agriculture, providing essential resources such as wool, meat, and milk [1]. Maintaining optimal reproductive performance and physiological health in sheep is critical for the sustainability and profitability of farming operations. Copper, an essential trace mineral, plays a pivotal role in numerous biochemical processes, including iron metabolism, collagen synthesis, and antioxidant defense mechanisms [2]. Although copper shortage causes health problems [3], excessive copper consumption can be toxic, especially for sheep [4]. Effective supplements depend on understanding the upper acceptable limits of copper consumption considering breed-specific variations in copper metabolism [5].

Copper absorption in ruminants is inherently low (<1.0–10.0%) compared to non-ruminants, primarily due to complex interactions within the rumen environment [5,6]. While copper is vital, sheep are uniquely sensitive to copper toxicity due to their limited ability to excrete excess copper via bile and their reduced capacity to store copper bound to metallothioneins in the liver [7]. Excess dietary copper intake can result in hepatic accumulation, leading to toxicity characterized by hepatitis, anemia, icterus, and hemoglobinuria [8]. Dietary copper requirements for sheep range from 4.3 to 28.4 mg/kg DM, depending on factors such as breed, age, and the copper source [9].

Breed differences significantly influence susceptibility to copper toxicity; for example, North Ronaldsay sheep are highly susceptible, whereas Merino sheep exhibit greater tolerance. Breeds such as Suffolk, Texel, Bluefaced Leicester, and Charollais are more susceptible compared to the copper-tolerant Cambridge breed [9]. Lambs are particularly vulnerable to copper toxicity due to their higher intestinal absorption rates [10]. Organic copper forms have garnered attention as dietary supplements due to their superior bioavailability and efficiency compared to inorganic forms. Although their potential is significant, previous study has investigated the acceptable upper limits of organic copper supplementation for Awassi lambs [11]. Preventing toxicity while maximizing mineral nutrition in small ruminant production depends on understanding these thresholds.

This study addresses a critical research gap by examining the effects of high organic copper supplementation on growth performance, mineral metabolism and organ histology in Awassi lambs. Given the breed’s susceptibility to copper accumulation, this study aims to determine the maximum tolerable limits of dietary copper while assessing potential risks to liver and kidney function. The findings will contribute to establishing optimal dietary copper supplementation for small ruminants, ensuring both nutritional benefits and long-term health. Additionally, this study provides valuable insights into the safe and effective use of organic copper in sheep farming, with implications for improving productivity, reproductive efficiency, and overall welfare.

## 2. Materials and Methods

The study was conducted at a sheep farm located in Haditha City, Anbar, Iraq, at coordinates 41°05′24.06″ E and 34°22′4.59″ N. All procedures adhered to the Animal Utilization Protocol approved by the Animal Experimentation Ethics Committee at the University of Anbar (Ref. 234L2024).

### 2.1. Animal Management and Experimental Feeding

Fifteen male Awassi lambs, with an average live weight of 16 ± 1.53 kg, were randomly assigned to three treatment groups (five animals per group). The first group (T1) served as the control and was fed the basal diet. The second (T2) and third (T3) groups were supplemented with copper in the basal diet at dosages of 0.5 g copper/kg DM and 1 g copper/kg DM, respectively. The basal diet was formulated to provide 2.4 Mcal/kg metabolizable energy and 14% crude protein, consisting of soybeans, wheat, and barley (Table 1). Organic copper was supplemented in the form of Copper proteinate 15% (Buffermin, JH Biotech Inc., Ventura, CA, USA), a chelated form of copper with amino acids and hydrolyzed proteins. Copper proteinate contains >25% crude protein and <15% copper. The total dietary copper concentrations were 6.18, 81.68, and 156.75 ppm in T1, T2, and T3 groups, respectively. Lambs were fed with a total mixed ration comprising wheat straw and feed concentrate (300 and 700 g/day, respectively) during a two-week adaptation phase.

The trial duration was 90 days. At the beginning of the trial, all animals underwent deworming, followed by a 14-day adaptation period. During the adaptation phase, all animals were fed the basal diet based on 4% equivalent of initial body weight without copper supplementation to allow for elemental depletion. All animals were housed individually in separate cages, and feeding occurred twice daily at 7:00 and 17:00. All animals were humanely slaughtered at the end of the trial after fasting for 20 h.

### 2.2. Body Biometric Measurement and Sample Collection

Body biometric measurements of each animal were recorded 24 h before the end of the trial to determine the effect of organic copper on growth. These measurements were obtained using flexible tape and vernier caliper according to the method described by Bautista-Díaz et al. [12]. Upon slaughter, blood samples (10 mL) were drawn from the jugular vein into collection tubes containing disodium ethylene diamine tetra acetic acid for real-time PCR analysis. Serum was separated by centrifugation (Hettich Rotina 380R, Hettich, Tuttlingen, Germany) at 13,000× *g* for 10 min and stored in vials at −20 °C until further analysis. Post-slaughter, right kidney, right testis, and right liver lobes were excised from the carcass. For histological analysis, tissue samples were rinsed with normal saline solution and fixed in freshly prepared 10% neutral-buffered formalin. The collected samples were kept at room temperature until further analysis. For mineral analysis, tissue samples were snap-frozen in liquid nitrogen and stored at −80 °C until further analysis.

### 2.3. Mineral Analysis

Mineral analysis was performed on the liver, kidney, and testes tissues following the procedure described by Saito [13]. One gram of sample was wet-ashed using 10 mL of concentrated nitric acid (HNO_3_, 98%) and 5 mL of perchloric acid (HClO_4_). Copper and zinc concentrations were determined using flame atomic absorption spectrophotometer (AAS700, PerkinElmer, Waltham, MA, USA) which has to assess the relationship as shared absorption and transport pathways between copper and zinc affect mineral homeostasis.

### 2.4. Histological Analysis

Tissue samples fixed in neutral buffered formalin were dehydrated using a graded series of ethyl alcohol, embedded in paraffin wax, sectioned at 5 μm, mounted on slides, and stained with hematoxylin and eosin. Images were captured using an upright light microscope equipped with a digital camera (Camedia C-5060, Olympus, Tokyo, Japan). For kidney samples, images were obtained from 10 slides under 40× magnification. The diameters of renal corpuscles, glomeruli, and the urinary space were measured following the method described by Rasool et al. [14]. Maximum and minimum diameters of renal corpuscles and glomeruli were recorded using the point-counting technique, and the average diameters were calculated using the following formulas:

$$Average\;diameter(µ)=\frac{Maximum\;diameter+Minimum\;diameter}{2}$$

$$Average\;Urinary\;space(µ)=\frac{Average\;renal\;corpuscular\;diameter-Average\;glomerular\;diameter}{2}$$

For liver samples, histological characteristics were quantified using the ImageJ/Fiji software (Version 1.54p, Bethesda, Bethesda, MD, USA), following the protocols outlined by Schneider et al. [15]. After calibration, features such as hepatic parenchymal swelling, vacuolated hepatocytes, necrotic cells, epithelial cell proliferation in bile ducts, and Kupffer cell activity were assessed. Measurements of regions were performed using the Polygon Tool, and cell counts were determined using the Cell Counter plugin (Version 2.2.2, Kurt De Vos, Bethesda, MD, USA). Four replicates were analyzed for each sample to ensure comprehensive evaluation of histological changes.

### 2.5. Gene Expression Analysis of Blood Serum

Total RNA was extracted from blood serum samples using the QIAamp RNA Blood Mini Kit (Qiagen, Hilden, Germany) following the manufacturer’s protocol. The quality and quantity of the extracted RNA were assessed using a NanoDrop ND-1000 spectrophotometer (NanoDrop Technologies, Wilmington, DE, USA). Primers were designed for three target genes, *IGF1*, *ATP7A*, and *ATP7B*, that focus on copper transport and metabolism, along with *GAPDH* as the housekeeping gene, using Primer3Plus software (Whitehead Institute for Biomedical Research, Cambridge, MA, USA) and *Ovis aries* sequences from the GenBank database (NCBI, Bethesda, MD, USA). The primers, manufactured by Macrogen company (Macrogen Inc., Seoul, Republic of Korea), are detailed in Table 2.

Quantitative real-time PCR (qRT-PCR) was performed using the CFX96 Touch Real-Time PCR Detection System (BIO-RAD, Hercules, CA, USA) with a total reaction volume of 25 µL. The reaction mixture consisted of 1 µL of each primer, 12.5 µL of SYBR Green Master Mix (Qiagen, Germantown, MD, USA), 9.5 µL of PCR-grade water, and 1 µL of cDNA. The qPCR conditions included an initial denaturation at 95 °C for 5 min, followed by 50 cycles of denaturation at 95 °C for 10 s, and annealing and extension at 60 °C for 30 s. A melting curve analysis was performed by increasing the temperature incrementally by 1 °C every 5 s from 65 °C to 95 °C. Amplification signals for each sample were normalized to *GAPDH* Ct values, and relative mRNA expression levels of the target genes were calculated using the 2^−ΔΔCt^ method. Each reaction was performed in triplicate to ensure accuracy and reproducibility.

### 2.6. Statistical Analysis

The effects of organic copper supplementation across the three treatment groups were analyzed using a completely randomized design (CRD) and subjected to analysis of variance (ANOVA). The model included treatment as a fixed factor, with individual animals serving as the experimental unit. Statistical analysis was performed using the GLM method in SAS (Version 9.4, SAS Institute, Inc., Cary, NC, USA). Mean comparisons were conducted using Duncan’s multiple range test at a 5% significance level following the identification of significant differences through ANOVA.

## 3. Results

The body biometric and testicular measurements of lambs across the three treatment groups are summarized in Table 3 and Table 4. Lambs fed the T3 diet exhibited significantly greater (*p* < 0.05) height at withers compared to the control (T1) and T2 groups, both of which positively correlated with body weight. Additionally, lambs in the T2 group showed significantly higher (*p* < 0.05) rump height compared to those in T1 and T3. No significant differences were observed among the groups for body weight, and other body biometric characteristics. Testicular length was significantly higher (*p* < 0.05) in the T3 group compared to T2 at the end of the trial (Table 4), while T1 showed no significant differences compared to the copper supplemented groups. However, no significant differences were observed among the treatment groups for other parameters, including scrotum length, width, height, circumference, thickness, and testicular width.

The concentrations of copper and zinc in the liver, kidney, and testes of lambs fed organic copper are presented in Table 5. Organic copper supplementation significantly increased (*p* < 0.05) hepatic copper concentration in proportion to the level of copper fed, compared to the control group (T1), with no similar effect observed in the kidney or testis. Conversely, zinc concentration was significantly lower (*p* < 0.05) in the kidneys of lambs in the T2 and T3 groups compared to T1. Both zinc and copper concentrations in the testes remained unaffected by the treatments.

**Figure 1:**
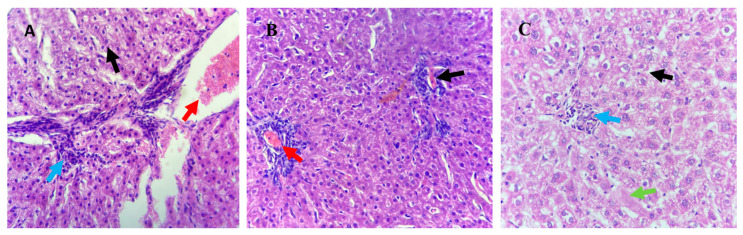
Histological analysis of liver tissues from Awassi lambs supplemented with organic copper. (**A**) Lambs supplemented with 0 g of organic copper (T1). (**B**) Lambs supplemented with 0.5 g of organic copper (T2). (**C**) Lambs supplemented with 1 g of organic copper (T3). The liver tissues were stained with hematoxylin and eosin and observed under 40× magnification using a light microscope.

Histological analysis on the liver, kidney and testes tissues was conducted to determine the effect of organic copper supplementation on these organs. No significant histological changes were detected in the longestioc and hepatic stellate cells with collagen fibrils across the treatment groups (Figure 1). Liver histopathology in the T1 group revealed hydropic degeneration in hepatocytes (black arrows). Similarly, the T3 group showed hydropic degeneration (black arrow) along with central necrosis (green arrow). Irregular inflammatory cell infiltration (blue arrows) and blood vessel congestion (red arrows) were observed in liver sections from all treatment groups (T1, T2, and T3).

**Figure 2:**
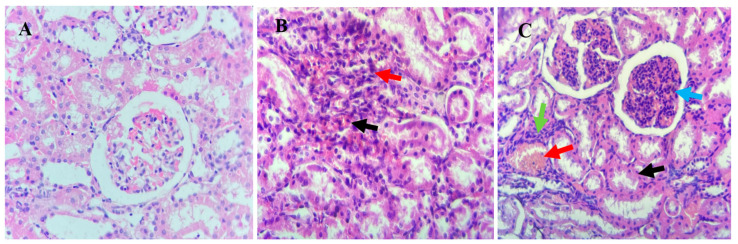
Histological analysis of kidney tissues from Awassi lambs supplemented with organic copper. (**A**) Lambs supplemented with 0 g of organic copper (T1). (**B**) Lambs supplemented with 0.5 g of organic copper (T2). (**C**) Lambs supplemented with 1 g of organic copper (T3). The kidney tissues were stained with hematoxylin and eosin and observed under 40× magnification using a light microscope.

In the kidneys, lambs from the T3 group exhibited marked tubular epithelial cell hydropic degeneration (black arrow), mesangial cell hyperplasia (blue arrow), blood vessel congestion (red arrow), and inflammatory cell infiltration (green arrow). Lambs in the T2 group showed interstitial hemorrhage (black arrow) and inflammatory cell infiltration (red arrow), while the kidneys of T1 lambs displayed normal histopathological features (Figure 2).

**Figure 3:**
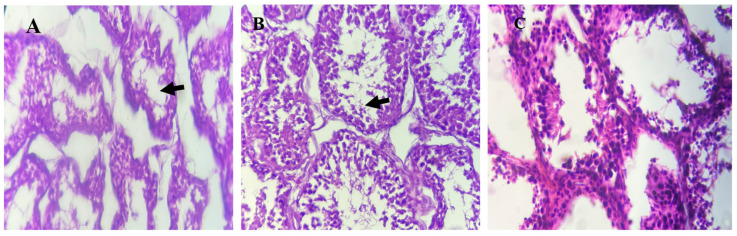
Histological analysis of testes tissues from Awassi lambs supplemented with organic copper. (**A**) Lambs supplemented with 0 g of organic copper (T1). (**B**) Lambs supplemented with 0.5 g of organic copper (T2). (**C**) Lambs supplemented with 1 g of organic copper (T3). The testes tissues were stained with hematoxylin and eosin and observed under 40× magnification using a light microscope.

Testes from lambs in the T1 group demonstrated normal histological architecture and active spermatogenesis (Figure 3). In contrast, T2 group testes exhibited spermatogenesis inhibition, and the T3 group showed mild spermatogenesis arrest, as indicated by black arrows.

The measurement of renal tubules’ diameter was recorded (Table 6). The study showed that the average diameter of the renal glomerulus in the control group and the treatment groups (T2 and T3) was 2.97, 3.98, and 3.17 mm, respectively. The data analysis revealed that there was no significant difference in the average diameter of renal glomerulus and renal corpuscles between the groups.

The diameter of renal tubules was assessed in the kidneys of lambs across the treatment groups. The study revealed no significant differences (*p* > 0.05) in the average diameter of renal glomeruli and renal corpuscles among T1, T2, and T3 groups. However, supplementation of organic copper significantly affected the liver histology of Awassi lambs, inducing notable pathological changes (Table 6). Lambs from T3 group exhibited a significant increase (*p* < 0.001) in hepatic parenchymal swelling, vacuolated hepatocytes, isolated parenchymal cell necrosis, and epithelial cell proliferation in the bile ducts compared to T1 and T2 groups. Conversely, the abundance of Kupffer cells was lower in the T3 group compared to the other treatments. These findings highlight the pronounced dose-dependent impact of organic copper supplementation on liver histopathology.

**Figure 4:**
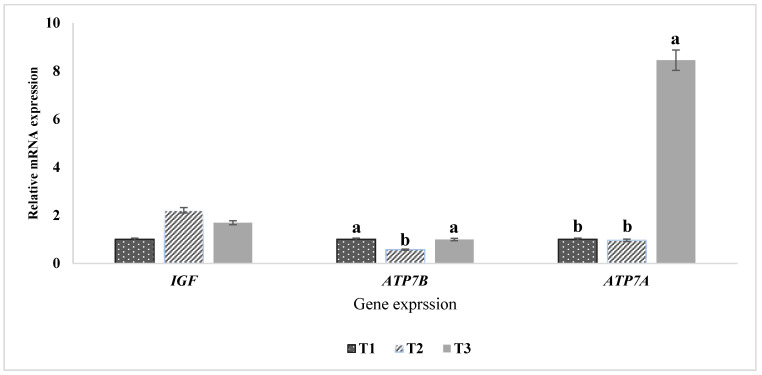
Expression of *IGF1*, *ATP7A*, and *ATP7B* genes in the blood serum of Awassi lambs. The mRNA expression levels of *IGF1*, *ATP7A*, and *ATP7B* were quantified using qRT-PCR, with GAPDH as the reference gene for normalization. Treatment groups include T1 (control: 0 g Cu/kg DM), T2 (0.5 g Cu/kg DM), and T3 (1 g Cu/kg DM). Bars with different letters (a, b) indicate significant differences (*p* < 0.05) among the groups.

The expression levels of *IGF1*, *ATP7A*, and *ATP7B* genes in the blood serum of Awassi lambs were evaluated (Figure 4). The data revealed that high dietary organic copper supplementation significantly (*p* < 0.05) increased *ATP7A* expression in the serum of T3 lambs compared to T1 and T2 groups. In contrast, the expression of the *ATP7B* gene in T2 was significantly lower than in T1 and T3 groups. No significant differences were observed in the expression of the *IGF1* gene among the treatment groups, indicating that dietary organic copper supplementation did not affect *IGF1* genotype expression. These findings suggest a specific regulatory role of organic copper on copper-related gene expression without influencing growth factor gene.

## 4. Discussion

Along with a balanced intake of protein and energy, DM consumption plays a critical role in determining the physiological growth of animals. Ensuring optimal nutrient intake is essential for improving growth performance and reproductive efficiency in Awassi lambs. Optimizing sheep production requires meeting these dietary needs while maintaining rumen health, particularly by sustaining a stable ruminal flora, which is important for metabolic efficiency. Organic copper supplementation enhances metabolic processes by improving enzymatic activity, supporting antioxidant defenses, and promoting efficient nutrient utilization. This study aimed to evaluate the impact of high dietary organic copper supplementation on growth performance, mineral metabolism, and organ histology in Awassi lambs. To assess these effects, lambs of similar initial weight were selected at the beginning of the study and fed a conventional feed ration set at 4% of their body weight [16,17].

The present findings align with previous studies that reported no significant impact on weight gain in lambs supplemented with copper at doses of 10 or 30 mg Cu/kg in the form of copper sulfate and copper-methionine [18] or copper proteinate [19]. Similarly, Eren et al. [20] found no significant effect on live weight when lambs were fed a basal diet supplemented with 5.25 mg/kg DM of organic copper chelate. These results suggest that while organic copper supplementation does not directly enhance growth performance, it may contribute to physiology adaptations that support metabolic stability in Awassi lambs.

This study identified a correlation between age, body weight, and body measurements in lambs across all treatment groups. Deribe et al. [21] highlighted a robust association between body weight and biometric parameters such as heart girth, chest depth, and height at the waist across various age groups. Costa et al. [22] further demonstrated that wither height could serve as a reliable predictor of carcass weight using biometric measurements. However, variations in sheep body measurements are greatly influenced by latitude and breeding conditions [23].

Exposure to elevated levels of heavy metals in contaminated environments has been shown to adversely affect the structure and function of the testes [24]. In contrast, this study found that high dietary organic copper supplementation had no detrimental effects on testicular growth or function. Testicular biometrics in lambs supplemented with organic copper were comparable to those in the control group, except for testicular length, which was significantly greater in the T3 group. This increase in testicular length suggests a potential benefit of organic copper in supporting testicular development and reproductive capacity. Testicular copper levels were similar among the groups, with no apparent adverse effects on testicular morphology. This suggests that testicular histology was not adversely affected by the absorption or bioavailability of organic copper. On the contrary, the observed increase in testicular length may indicate enhanced spermatogenesis, which could improve reproductive efficiency in Awassi lambs.

Copper plays a vital role in various physiological processes, including synthesis of collagen and antioxidant enzyme activity, both of which improve tissue integrity and function. Previous studies have linked copper deficiency to impaired testicular development and seminiferous tubule damage [20,25]. Martínez et al. [26] reported that testicular weight directly affects daily sperm production and sperm concentration in rams, with an average production rate of 20 million sperm per gram of testis per day. Thus, the increase in testicular length could indicate improved spermatogenic capacity, ultimately enhancing the reproductive efficiency. This finding underscores the potential role of copper in male fertility, warranting further investigation into its impact on semen quality. Consistent with our findings, Awassi ram lambs displayed similar testicular width and thickness, indicating a strong correlation between body weight and growth patterns. Furthermore, studies have demonstrated that supplementation with both zinc and copper resulted in a steady, dose-dependent increase in all scrotal biometric parameters from the 38th week onward [27,28].

Mineral analysis in the present study reveals that copper concentrations in the liver increased in lambs supplemented with organic copper in the T2 and T3 diets, whereas kidney copper levels remained within the normal range of 6–279 ppm [29]. This indicates that liver copper accumulation is a more reliable indicator of copper status, particularly during hemolytic crises, than kidney copper levels. The lower liver copper concentrations observed in T1 may be attributed to the lower dietary copper intake, whereas the higher levels observed in T2 and T3 align with previous study, confirming the impact of supplementation [30]. The increased hepatic copper levels suggests that Awassi lambs possesses robust homeostatic mechanisms that regulate copper storage and prevent excessive accumulation in vital organs, contributing to their adaptability to varying dietary copper levels. This resilience is supported by previous findings that copper toxicity in sheep typically involves liver concentrations of 1000–3000 ppm and kidney levels of 70–200 ppm during hemolytic crises [31].

Zinc dynamics exhibited significant changes, particularly in kidney zinc concentrations, which decreased in T2 and T3 lambs. This reduction may be attributed to the increased synthesis of metallothionein in mucosal cells, stimulated by the high dietary copper levels. Zinc binds to the metallothionein transcription factor (MTF-1), which limits its absorption and indirectly affects copper metabolism [32]. This interplay between copper and zinc metabolism highlights the importance of considering trace mineral interactions when formulating dietary supplements for livestock.

Histological analyses of the kidney showed no necrosis or mortality in any treatment group, indicating the non-toxic nature of organic copper supplementation. Mild tubular epithelial cell degeneration and inflammatory infiltration were observed in T3 lambs, while T2 lambs exhibited interstitial bleeding. These findings align with earlier studies that linked hemolytic crises to kidney damage characterized by tubular injury and the presence of hemoglobin deposits [33]. Liver histology revealed abnormalities across all groups, including inflammatory cell infiltration and vascular congestion, with more pronounced changes in T3 lambs. Despite these observations, the absence of clinical toxicity underscores the resilience of the Awassi breed, which appears to effectively regulate hepatic copper elimination to mitigate systemic toxicity [9]. Kupffer cells, though reduced in T3 lambs, likely played a key role in immunological monitoring and repair, limiting further hepatic damage [34].

Although present findings indicated liver damage in the T3 group with only 276.44 ppm of accumulated copper, systemic toxicity was not observed with the supplementation of 1 g Cu/kg DM. This suggests that while Awassi lambs exhibit some degree of resistance to copper toxicity, their threshold for hepatic copper accumulation may be lower than previously assumed [35]. While Awassi sheep are generally considered more resistant to copper toxicity than certain other breeds, this study did not include a direct comparison with another breed receiving the same level of supplementation. Further comparative research involving different breeds is necessary to determine whether the physiological responses to copper supplementation are due to a general adaptation mechanism or breed-specific sensitivity [36,37].

Essential trace elements play a pivotal role in regulating various physiological functions, including the transport of reactive oxygen species, DNA synthesis, cellular signal recognition, and nutrient metabolism [38]. Among these, copper transport is mediated by the *ATP7A* and *ATP7B* genes, which encode P-type Cu-ATPases responsible for facilitating copper transport to ceruloplasmin and lysyl oxidase, as well as exporting excess copper from cells [39]. Our findings revealed a significant upregulation of *ATP7A* and *ATP7B* genes in lambs supplemented with 1 g/kg DM organic copper (T3), suggesting an adaptive cellular response aimed at regulating copper homeostasis.

The increased expression of *ATP7A* and *ATP7B* genes in the T3 group mirrors observations in copper-resistant Chinese hamster ovary cells, where *ATP7A* gene amplification led to higher endogenous *ATP7A* levels and an enhanced capacity to export copper [29,40]. Such elevated expression is known to confer copper resistance in cells, serving as a functional assay for evaluating the role of copper-transporting P-type ATPases and the impact of their mutations [41]. In contrast, the expression of the *IGF1* gene, known for its diverse biological effects, did not differ significantly across the treatment groups. *IGF1* plays a critical role in regulating normal growth, development, immunity, and metabolism in vertebrates [42,43]. The lack of *IGF1* expression regulation in this study suggests that, under normal physiological conditions, *IGF1* activity in sheep may not be influenced by dietary copper levels. This further supports the idea that copper metabolism is tightly regulated in Awassi lambs without significantly affecting growth-related endocrine pathways.

## 5. Conclusions

High dietary organic copper supplementation had no significant effect on growth performance or body biometric measurements in Awassi lambs. Despite increased hepatic copper accumulation, kidney copper levels remained within normal ranges, suggesting efficient homeostatic regulation. The observed increase in testicular length in lambs receiving 1 g Cu/kg DM suggests potential benefits for spermatogenesis by enhancing testicular development, without adverse effects on testicular histology. However, mild histopathological changes in the liver and kidneys, particularly in the highest supplementation group (T3), indicate a potential threshold for copper tolerance in Awassi sheep that requires further exploration. The upregulation of *ATP7A* and *ATP7B* genes in response to higher copper levels demonstrate an adaptive physiological mechanism for copper transport and cellular adaptability. Meanwhile, *IGF1* expression remained unchanged, suggesting that copper supplementation did not influence growth-related genetic activity. Although Awassi sheep are traditionally considered resistant to copper toxicity, the histological findings suggest that their susceptibility to copper-induced liver damage may be greater than previously assumed. These findings contribute to a deeper understanding of mineral supplementation in sheep, emphasizing the Awassi breed’s robust copper regulation mechanisms while highlighting the need for further studies to define safe supplementation limits that optimize health and productivity.

## Figures and Tables

**Table 1:** Ingredients and chemical compositions of different supplementation of organic copper into experimental diets (DM basis).

Dietary Ingredient (g/kg)	T1	T2	T3
Wheat straw	300	300	300
Rolled barley	400	399.5	399
Cracked wheat	150	150	150
Soyabean meal (44%)	120	120	120
Copper proteinate	0	0.5	1
Limestone	10	10	10
Salt	10	10	10
Vitamin–mineral mix	10	10	10
Total	1000	1000	1000
Chemical composition (%)			
DM	90.0	90.2	90.5
OM	95.0	94.5	94.0
Ash	5.0	5.5	6.0
CP	14.0	14.5	14.2
EE	2.5	2.3	2.2
CF	13.0	12.8	12.5
Cu (ppm)	6.18	81.68	156.75

Vitamin–mineral premix: vitamin A, 10,000,000 IU; vitamin E, 70,000 IU; vitamin D, 1,600,000 IU; calcium, 2%; phosphorous, 1%; magnesium, 12.0%; chloride, 20 mg/kg; sodium, 25%: selenium, 90 mg/kg; cobalt, 150 mg/kg; iodine, 3000 mg/kg; manganese, 3000 mg/kg; zinc, 4000 mg/kg. DM: dry matter; OM: organic matter; CP: crude protein; EE: ether extract; CF: crude fiber.

**Table 2:** Sequences of primers for quantitative real-time polymerase chain reaction (qRT-PCR) in blood serum of Awassi lambs.

Genes	Primer Sequences (5′–3′)	Annealing Temperature (°C)	Accession Number
*IGF1*	F: CAGGAGCACGAGAGGAAGAGA R: GATGGTACGTGACAAGGCAGG	79	XM_060411608.1
*ATP7A*	F: AAGAGGAGGGGAAACGGGTAG R: GCTGCTTCAATGGCTACGTCT	83	XM_004022208.6
*ATP7B*	F: GGCAGTCATCACTTACCAGCC R: CGTCTATGGGTCCCAGGCTTA	79	XM_060394019.1
*GAPDH*	F: ACCACTTTGGCATCGTGGAG R: GGGCCATCCACAGTCTTCTG	61	XM_060411595.1

**Table 3:** Effect of different levels of organic copper supplementation on body biometrics characteristics (cm) of Awassi lambs.

Parameters	Body Weight (kg)	Height at Withers	Rib Depth	Body Diagonal Length	Body Length	Pelvic Girdle Length	Rump Depth	Rump Height	Pin Bone Width	Hook Bone Width	Abdomen Width	Girth Circumference	Abdomen Circumference
T1	33.66	64.00 ^b^	19.66	94.66	36.33	19.00	28.00	55.00 ^b^	23.33	24.00	43.00	51.33	99.00
T2	29.75	61.50 ^b^	16.50	88.33	31.66	17.33	25.33	64.00 ^a^	27.66	25.00	43.66	56.00	93.66
T3	35.89	67.00 ^a^	23.00	92.50	35.00	21.00	22.00	62.00 ^b^	30.00	28.50	44.50	45.00	96.50
SEM	1.36	1.02	1.24	2.38	1.12	0.98	1.12	1.80	2.10	0.89	0.94	1.87	2.12
*p* value	0.1917	0.0236	0.2219	0.5850	0.1970	0.4168	0.0994	0.0236	0.5721	0.0526	0.8697	0.1177	0.6310

T1: (control; 0 g Cu/kg DM), T2: (0.5 g Cu/kg DM), and T3: (1 g Cu/kg DM). ^a,b^ Means in the same row with different superscripts differ significantly at *p* < 0.05.

**Table 4:** Effect of different levels of organic copper supplementation on testicular dimensions (cm) of Awassi lambs.

Parameters	Scrotum Length	Scrotum Width	Scrotum Height	Scrotum Circumference	Scrotum Thickness	Testicular Length	Testicular Width
T1	9.90	8.84	31.33	24.66	0.34	7.73 ^a,b^	4.55
T2	10.91	7.90	27.66	24.66	0.33	6.20 ^b^	3.98
T3	9.29	7.73	26.50	26.00	0.36	8.99 ^a^	4.09
SEM	0.38	0.35	1.38	0.85	0.02	0.49	0.17
*p* value	0.2434	0.4592	0.3909	0.8116	0.8553	0.0498	0.4921

T1: (control; 0 g Cu/kg DM), T2: (0.5 g Cu/kg DM), and T3: (1 g Cu/kg DM). ^a,b^ Means in the same row with different superscripts differ significantly at *p* < 0.05.

**Table 5:** Effect of different levels of organic copper supplementation on mineral concentration (ppm) in liver, kidney, and testes of Awassi lambs.

Tissue	T1	T2	T3	SEM	*p* Value
Liver					
Cu	26.95 ^b^	192.44 ^a^	276.44 ^a^	30.121	0.001
Zn	99.34	93.56	106.34	4.143	0.5434
Kidney					
Cu	20.98	15.76	21.00	1.700	0.4005
Zn	102.24 ^a^	72.84 ^b^	95.64 ^b^	5.528	0.0434
Testis					
Cu	12.51	16.17	10.12	1.212	0.0574
Zn	95.42	95.62	96.06	1.643	0.9893

T1: (control; 0 g Cu/kg DM), T2: (0.5 g Cu/kg DM), and T3: (1 g Cu/kg DM). ^a,b^ Means in the same row with different superscripts differ significantly at *p* < 0.05.

**Table 6:** Effect of organic copper supplementation on renal tubule and glomerular diameters, and liver histology in Awassi lambs.

Parameters	T1	T2	T3	SEM	*p* Value
Renal glomerulus diameter (mm^2^)					
Maximum	3.40	4.03	3.43	0.470	0.8514
Minimum	2.55	3.93	2.92	0.398	0.3775
Mean	2.97	3.98	3.17	0.380	0.6392
Renal corpuscle diameter (mm^2^)					
Maximum	4.30	3.30	2.92	0.290	0.2044
Minimum	2.95	3.06	2.15	0.426	0.4122
Mean	3.63	3.18	2.39	0.329	0.3258
Hepatic parenchymal swelling (mm^2^)	0.60 ^b^	0.87 ^b^	2.45 ^a^	0.246	0.001
Hepatocytes with vacuolated appearance (mm^2^)	0.11 ^b^	0.67 ^b^	1.25 ^a^	0.141	0.001
Necrosis of isolated Parenchymal cells (mm^2^)	0.34 ^b^	0.13 ^b^	0.57 ^a^	0.056	0.001
Proliferation of epithelial cells of bile ducts (mm^2^)	0.21 ^b^	0.47 ^a,b^	0.86 ^a^	0.081	0.001
Numerous kupffer cells (Cell)	15.00 ^a^	16.50 ^a^	11.50 ^b^	0.699	0.001

T1: (control; 0 g Cu/kg DM), T2: (0.5 g Cu/kg DM), and T3: (1 g Cu/kg DM). ^a,b^ Means in the same row with different superscripts differ significantly at *p* < 0.05.

## Data Availability

The data that support the findings of this study are available from the corresponding author upon reasonable request.
